# Leveraging data analytics to enhance public health system performance in Peru

**DOI:** 10.3389/fpubh.2026.1849983

**Published:** 2026-07-17

**Authors:** Edmundo Lizarzaburu, Julio Quispe, Macarena Lizarzaburu, Fidel Amesquita, Fausto Lizarzaburu

**Affiliations:** 1 Esan University, Lima, Peru; 2 Monash University, Melbourne, VIC, Australia; 3 Universidad de Lima, Lima, Peru

**Keywords:** big data, digital health, health equity, Peru, predictive analytics, public health

## Abstract

The public health systems in Latin America are being transformed and restructured with the help of digital technologies and the use of Data analytics. This transformation is occurring in countries where there is a significant imbalance between the amount and quality of health care services provided, as well as between the institutions that provide these services, particularly in Peru. The increased use of digital health technologies, predictive analytics, and interoperable information systems in Peru provides an opportunity for the improvement of the overall performance of Peru’s Public Health System through the use of digital health technologies and predictive analytics and therefore also the ability to allocate resources in a more equitable manner and provide improved epidemiological surveillance. In this review, the author examines the role of data analytics in strengthening Peru’s public health systems, as well as the main structural, regulatory, and technology barriers inhibiting data analytics’ implementation in Peru’s public healthcare systems. A literature review was conducted using readily accessible peer-reviewed articles contained within institutional and governmental publications as well as international health governance documents found in Scopus, PubMed, SciELO, Web of Science, and Google Scholar from 2014 through 2026 for the subject areas of digital health, predictive analytics, interoperability, the use of artificial intelligence, and the ways in which these technologies affect the future of public health governance in Peru and throughout Latin America. The research highlights that analytics are improving how chronic diseases are monitored, how quickly diseases are detected, as well as improving the decision-making abilities of health systems and governments across multiple countries within Latin America. Additional barriers still exist in Peru; these include limited interoperability between healthcare organizations, unevenly distributed technology infrastructure, fragmented governance structures, an inadequate amount of technical resources, and continuing differences in the level of provision of technological resources in urban vs. rural areas. In addition, it was identified through the review that the mere adoption of technology alone will not result in a change; rather, it is essential that there be good institutional coordination for implementing and overseeing regulatory practices, ethical governance of the use of this technology, and development of a strategy for ensuring inclusiveness of implementation. Through its proposed integrated analytical framework, this study expands the public health literature by demonstrating how interoperability (including the impact of governance), predictive analytics and equity dimensions of healthcare systems are related to public health systems in Peru. Also, based on findings from this research, public health systems will need to invest for the long term in improving their infrastructure, creating modern regulatory schemes for digital health, training their workforce in using these tools, and developing policies designed to provide digital inclusion to all members of the community if they want to achieve a sustainable digital transformation.

## 1 Introduction

The public health systems in Latin American countries are quickly going through a major change that makes use of new technology to take advantage of data analytics, artificial intelligence, electronic health records, and interoperable health information systems (1, 2). These technologies have been increasingly recognised as strategic tools by government agencies for epidemiological surveillance, operational efficiency in allocating healthcare resources, support for clinical decision making, and increased access to healthcare for vulnerable populations (3, 4). As a result of data-driven public health management being a priority for emerging economy governments, they frequently have issues with fragmentation of the healthcare delivery system, limited funding, and territorial inequities (5, 6).

Peru is an important example of how this process of change is taking place throughout the region. While some progress has been made over the years to expand the availability of health care to citizens throughout the country, there remain numerous issues with the Peruvian Public Health System that inhibit access to care for many people (7, 8). Some of these issues have been the result of a historically fragmented health care system that includes a number of different entities providing health care services; e.g., the Ministry of Health (MINSA), EsSalud, regional governments, and private healthcare providers. This fragmentation has made it difficult to facilitate communication and collaboration between the various entities providing health care services in Peru (9, 10). The lack of integration between these entities became even more apparent during the COVID-19 Pandemic as many of the same issues that existed prior to the Pandemic were exposed related to data integration, real time surveillance, and limitations to institutional coordination (11, 12).

New possibilities have emerged as a result of advances in technology to overcome these systemic constraints. Predictive analytics, machine learning, digital health platforms, and currently available interoperable electronic health records all offer better opportunities to detect diseases earlier, utilize healthcare resources more effectively, monitor higher-risk population groups more effectively, and develop policies based upon objective evidence (13, 14). Some of the digital health and analytics projects in several Latin American countries are examples of how countries can use digital health and analytics to improve the effectiveness of public health systems, including using an interoperability standard framework, creating a telemedicine network, and using AI-assisted epidemiological surveillance tools (1, 15); however, the use of these technologies has also provided new opportunities to identify and address issues of governance, ethics and equity concerning issues such as data privacy, organizational capacity, algorithmic discrimination, and the digital divide (16, 17).

There is a considerable degree of disparity regarding the development of analytics-driven public health systems across Peru, and significant obstacles remain with regards to both the development of digital infrastructure and the availability of skilled personnel and resources, codifying intergovernmental relationships, and ensuring a robust national commitment to long term policy implementation (18, 19). In Peru, recent advancements in digital health modernization are a result of a combination of existing interoperability initiatives through MINSA, as well as an increased focus on developing and implementing technical standards for the exchange of health information, and efforts to engage in international discussions regarding the governance of AI and digital health (17, 18). However, the implementation of analytics-driven public health systems continues to be fragmented and inconsistent across the various geographic and institutional jurisdictions within Peru. Significant barriers continue to exist in the areas of digital infrastructure, technical capacity, governance coordination, and policy continuity (7, 20).

The purpose of this study is to assess how data analytics may be utilized to improve public health system performance in Peru, while also identifying the major structural, institutional and technical hurdles impeding implementation. The review focuses on four related areas of analysis: Interoperability, Predictive Analytics Applications, Governance Frameworks, and Health Equity; and provides a contribution to the body of knowledge regarding Public Health Transformation in Peru and Latin America by providing an Integrated Analytical Framework that explains how each of these Elements of a Digital Public Health Transformation collectively determine the sustainability and effectiveness of such transformation.

## 2 Methods

### 2.1 Review design

This research used a narrative literature review method to explore how data analysis may help increase the effectiveness of health-related programs in Peru and other countries throughout Latin America (1, 2). The focus of this review encompassed the relationship among digital health, predictive analytics, interoperability in digital health systems, governance, and social equality regarding access to healthcare services (4, 6). Although a complete system review was not performed, the researchers employed a systematic approach to searching the literature and synthesizing the results for improved clarity and consistency in methodology (7, 21).

### 2.2 Search strategy

A literature review that occurred from March through April 2026, drew from a variety of different university and other academic database sources, such as Scopus, PubMed, SciELO, and Google Scholar (which also includes articles published on these sites). Additional search results were obtained by searching for relevant national and International (WHO, PAHO, and MINSA) institutional and policy documents. These additional documents helped provide insights into the regulatory and governance perspective providing support for the digital Health Transformation process in Perú.

The search process employed combinations of the following keywords: “public health analytics,” “digital health,” “Peru,” “predictive analytics,” “electronic health records,” “health interoperability,” “artificial intelligence in healthcare,” “health governance,” and “Latin America.” Boolean operators were used to refine search combinations and improve relevance.

### 2.3 Inclusion and exclusion criteria

The literature search consisted of reviewing peer-reviewed journal articles, reports from institutions related to digital health system(s) and health analytics, interoperability between systems, artificial intelligence applications, and public health governance in Peru or Latin America from the years 2014 through 2026. Research related only to clinical medicine and without any relevance to public health systems or the management of health were disqualified. Also disqualified were editorials, abstracts submitted for presentation at conferences, duplicates, and non-academic opinion pieces.

### 2.4 Data synthesis

The literature review utilized a thematic synthesis methodology for analysis of the literature selected. A total of four analytic dimensions emerged from the analysis: Health Information Systems & Interoperability, Predictive Analytics & Epidemiological Surveillance, Governance & Regulatory Frameworks, and Equity & Digital Inclusion, which organised the review into dimensions that supported identification of barriers, challenges, and opportunities related to the use of analytics in Public Health Systems in Peru.

## 3 Results and literature synthesis

### 3.1 Interoperability and digital fragmentation

The need for interoperability continues to be one of the key obstacles to achieving a successful digital transformation of all areas of public health in Peru and Latin America, as indicated by many studies (9, 10). Interoperable health informatics systems and electronic health records (EHRs) are viewed as critical elements that will allow healthcare providers to coordinate activities more effectively with one another and exchange data more easily, and will help support evidence-based decision making in public health-related areas (15, 22). However, the available evidence shows that the various organizations and entities involved in providing healthcare to the people of Peru have not all developed their own systems for interoperability. As a result, implementation of such systems is limited and fragments across Peru’s different healthcare provider entities, including MINSA, EsSalud, the various regional healthcare systems, and the private sector (7, 18).

Over the past few years, many region-specific extensions have focused on improving the interoperability of health information systems by creating national strategies, integrating various types of systems, and implementing the appropriate national electronic systems (1, 2). One such example is Peru’s Ministry of Health, which has actively been promoting initiatives for improving interconnectivity between electronic health records (EHRs) and health information exchanges (HIEs) by establishing technical standards for both of these types of systems (18). Additionally, the regional collaborative efforts of the Pan-American Health Organization (PAHO) and the World Health Organization (WHO) have stressed the significance of using standardised health information systems to facilitate continuity of care, increase the number of instances of detection and interruption of communicable diseases (also known as epidemiological surveillance) and enhance the ability of public health organisations to plan for future health care needs (1, 2).

While there has been much progress made, there still remains a large gap between urban versus rural healthcare organizations when it comes to their ability to utilize technology, use the internet, and implement technology across the board (11, 16). Many of the rural healthcare organizations continue to operate as siloed entities with little or no ability to share data with one another; as such, the effectiveness of the analysis produced by analytics helps guide public health decisions is diminished (5, 20).

### 3.2 Predictive analytics and epidemiological surveillance

The increasing reliance on predictive analytics, big data, and artificial intelligence technologies has broadened the scope of healthcare systems to utilize their capabilities to enhance epidemiological surveillance, chronic disease management, and optimal use of Healthcare Resources (3, 4). In past studies in Latin America, Predictive Algorithms and Data Driven Decision Support Systems have demonstrated the potential to facilitate earlier detection of Disease, more effective Health Care Worker Utilisation and Enhanced Monitoring of At-Risk Groups (14, 15).

Several different technological advancements have been made recently, including using machine learning models in epidemiology; using GIS technology for monitoring the spread of infectious diseases over large geographical areas; and creating digital dashboards to visualize real-time surveillance of outbreaks (12, 13). In Peru, these technologies have become increasingly important due to the rapid development of integrated health information systems and the adoption of data-driven approaches to public health, particularly in response to COVID-19 outbreaks (8, 19).

Predictive analytics cannot be used effectively without having accurate, reliable, and available data again distributed across many healthcare organizations with different systems that do not talk to each other or work together properly (9, 10). Thus far, a lack of cohesive interoperability between these disparate database systems has limited the overall potential for analysing vast amounts of healthcare-related data throughout the entirety of Peru’s healthcare landscape (18, 20). Additionally, without a standard governance framework for Artificial Intelligence (AI) implementations and health data management, new challenges will continue to arise throughout the process of implementing analytics solutions in order to understand population health issues in Peru as well as predicting future public health outcomes (6, 17).

### 3.3 Equity and digital inclusion

One of the main areas of focus for Digital Public Health Transformation in Peru is Health Equity (1, 2). Digital health technology has the potential to increase access to Healthcare Services and improve the efficiency of how services are provided; however if the way that Digital Technologies are implemented does not consider the differences between territories, the socioeconomic status of individuals, and the access to technology, these differences may be accentuated and create even greater disparities (5, 16). Previous research has indicated that many marginalised and rural populations have less ability to connect with others digitally as well as less ability to access Digital Healthcare Services (11, 23, 24).

In Peru, because of unequal access to telecommunication services (in particular), geographic dispersion of location and institutional limitations of services in a remote region, the level of rural–urban inequality in urban areas is especially high (7, 8). For this reason, digital health systems and predictive analytics may be used most effectively in the urban areas of Peru if inclusive implementation policies are adopted (1, 2).

According to researchers, it is essential to develop an equitable system for governing digital reform efforts in health systems (6, 17). As noted above, equity-oriented governance frameworks include investments in digital infrastructure, educational programs for health care providers and other personnel who serve clients, community-level digital literacy initiatives, and policies to ensure equal access to interoperable services provided to vulnerable populations (1, 11).

### 3.4 Governance, regulation, and institutional coordination

As a result of increased interest in digital public health in Latin America, governance and regulations frameworks are becoming more necessary than ever (1, 2, 25). According to WHO and PAHO, digital transformation of the health sector necessitates not only technological improvement but also strong institutional governance/coordination, ethical governance and data protection (6, 17). Therefore, all systems for healthcare analytics must be implemented under a framework that ensures privacy protection, interoperability, transparency, accountability and equitable access to digital health services (1, 9).

Peru has recently made progress with digital health initiatives encouraging greater focus on Interoperability (I-OS) and Health Information Governance (HIG) by the Government (MINSA) (8, 18, 26). In terms of digitalization, MINSA is moving forward with the development of national standards, an electronic clinical record (ECR) implementation project, and national interoperability guidelines to open communication within the healthcare delivery ecosystem (18, 19). Also, the country has participated in and assisted in global discussions around Artificial Intelligence (AI) and the creation of responsible digital health regulations as part of regional collaborative projects supported by the Pan-American Health Organization (PAHO) and the World Health Organization (WHO) (1, 17, 27).

While advancements have been made, there are still many unresolved issues related to governance. The fragmentation of institutions dealing with health care delivery systems, inconsistent enforcement of regulations, limited coordination of technical standards, and inadequate long-term funding mechanisms impede progress on the large scale implementation of digital health solutions (7, 20). As demonstrated by several pilot studies that validated a concept or new technology within the context of a pilot study, many entered into contract negotiations with a Ministry of Health in order to expand their program, yet failed to successfully integrate them into a long-term plan for sustainable national integration (15, 21).

The lack of universal standards for Digital Health Architecture and Data Exchange also makes it difficult to integrate analytics into the entire Peruvian Healthcare system (9, 10). These limitations decrease the effectiveness of the use of Predictive Analytics Applications and impair the ability of institutions to provide coordinated public health responses to epidemiological emergencies (12, 14).

### 3.5 Evidence from quantitative and qualitative studies

Numerous approaches have been used in the literature reviewed to assess the implementation and effectiveness of digital health systems and digital health system analytics applications for the public health sector (including but not limited to: statistical analysis, predictive modelling, descriptive statistics) (4, 14). For the quantitative studies reviewed in the literature, the majority focus on the effect of predictive analytics on improving health care efficiencies, monitoring of epidemiological data, and the most appropriate use of resources (3, 12). Prior to this review, the literature reported that predictive analytics and electronic health records (EHR) have significantly enhanced the management of chronic diseases, increased the ability to detect outbreaks at an early stage, and improved the co-ordination of health services (15, 22).

Unlike quantitative studies that focus on the effectiveness of technologies in terms of their use, quantitative studies emphasize the impact of organisational and institutional barriers that hinder the implementation of technology (20, 21). As an example, resistance to new technology is one of the most frequently cited barriers to implementing new technology. Other commonly cited barriers include poor training for staff, a lack of co-ordination between institutions within a health care facility and differences in the quality of digital capability at health care facilities (7, 11). Qualitative studies also highlight the key roles of government structures, culture and political will in determining the long-term viability of a technology-based transformation (6, 17).

Mixed methods research combines qualitative and qualitative approaches to better understand the implementation of health technologies. By combining quantitative assessments of the effectiveness of health technologies with qualitative evidence about the feasibility of the technology in an institution, stakeholder perceptions of the technology, and the barriers to operationalizing the technology, a more comprehensive view of digital health technology implementation can be obtained (4, 21). Research literature on this subject indicates that successful public health analytics implementation is contingent upon both the technology’s ability to function - and the ability to adapt the institution, the quality of governance, and the consistency of implementation strategy to the needs of the community (1, 2).

A review of the current literature indicates that there have been many gaps in the implementation of digital transformations within the Public Health System in Peru between various institutions and geographies (8, 19). Within many areas of digital transformation in Public Health, including areas such as telemedicine, electronic health records, Epidemiological Surveillance and Predictive analytics, there has been recognition of tremendous successes (14, 15). However, there continue to be major structural barriers limiting scalability and long-term sustainability of the successful implementations. The data consistently indicate that fragmented Governance structures and unequal distribution of Digital Infrastructure, along with the existence of Interoperability limitations, and a continued existence of Social Inequalities represent significant challenges to the effective deployment of Analytics-Driven Public Health Systems throughout Peru (5, 20).

At the same time, evidence shows that Peru has a major opportunity to strengthen the effectiveness of the public health system through the combination of analysis technology, standards for interoperability, and digital governance (2, 18). The successful application of these technologies will necessitate coordinated institutional reforms; long-term financial investments; modernizing regulatory frameworks; and developing equity-based digital inclusion policies to help equalize the geographic differences in healthcare access and technological capabilities (1, 6) (see Table 1).

**Table 1:** Selected digital health and analytics initiatives in Latin America.

Country	Initiative	Main technology	Main contribution	Key barrier
Peru	Electronic Clinical Records and Interoperability Initiatives	EHR and interoperability systems	Improved health information exchange	Institutional fragmentation
Brazil	SUS Digital Programs	Big data analytics and telehealth	Expanded epidemiological monitoring	Governance complexity
Chile	National Telemedicine Systems	Remote healthcare platforms	Increased rural healthcare access	Connectivity gaps
Colombia	Digital Public Health Monitoring	Predictive analytics dashboards	Improved disease surveillance	Data integration limitations
Regional (PAHO/WHO)	Digital Health Governance Frameworks	AI governance and interoperability standards	Regional policy coordination	Uneven implementation capacity

Source: Own elaboration.

## 4 Discussion

This review has shown that successful implementation of analytics-driven public health systems in Peru requires interaction among the four areas of technological capacity, governance, and implementation strategies (equity-oriented) (1, 2). Most prior studies have focused on the technological potential of predictive analytics, electronic health records, and artificial intelligence, but our review indicates that without the proper interaction between technological innovations, issues of institutional governance, interoperability, and equity-oriented implementation strategies will not produce sustainable enhancements to the performance of public health systems (3, 6).

In Peru, Institutional fragmentation, unequal distribution of digital infrastructure, and lack of interoperability continues to limit the ability of many digital health initiatives to grow, even though they are undergoing modernization efforts (18, 20). The same will be true for many digital health programs within other Latin American health care systems; while pilot digital health projects often illustrate the technical feasibility of a digital project, they also have challenges related to achieving a national level of integration and therefore long-term sustainability (15, 21).

The research suggests that governance quality is one of the strongest determinants of whether or not an organisation will succeed with digital transformation (6, 17). Regulatory coordination, ethical oversight, data protection mechanisms, and institutional continuity are all necessary to ensure the operational effectiveness of both the organisation itself and its members, as well as to build public trust with the organisation (1, 9). Without these governance mechanisms, analytic systems may result in exacerbating existing social inequalities by concentrating the benefits of technology on urban or resource-rich healthcare organizations while excluding the most disadvantaged populations from these benefits (5, 16).

The evidence reviewed indicates that the use of analytics can enhance public health system performance within Peru, through the enhancement of epidemiological surveillance, evidence-based decision making, the optimization of resource allocation and increased monitoring of at-risk populations (4, 14). Previous studies completed in various countries throughout Latin America, suggest that predictive analytic tools, digital health platforms, and interoperable information systems will help healthcare organizations manage healthcare supply chains in a more efficient manner and improve institutional response times during a public health emergency (12, 15).

Analytical health care systems, using data for decision-making effectiveness, show that the effectiveness of such systems are significantly affected by the quality of governance, interoperability capabilities, institutional alignment, and the equitable implementation of strategies (1, 2). These findings indicate how these many factors interact with analytical health care systems’ performance. In the context of Peru, factors such as continued separation between health care institutions, differences in technological infrastructure and geographic-level disparities inhibit digital transformation optimization (8, 19).

The importance of combining technological innovations with an effective governance framework and appropriate policies is emphasized across the literature in order to make solid progress towards the goals of successful digital transformation within public health (6, 17). Therefore, institutions such as WHO and PAHO have stressed the importance of developing ethical governance frameworks to support the protection of patient privacy, improve data security and facilitate interoperability within health systems (1, 17). When such frameworks do not exist, digital health tools might yield unintended outcomes related to inequality in access, distrust in institutions, and lack of coordination during the implementation of initiatives (5, 11).

Efforts to modernize Peru’s health information systems, including interoperability standards, are a significant step forward in creating a more integrated public health infrastructure; however, long-term sustainability will require coordination among organizations at multiple levels, stable funding sources, increased technical capacity, and a reduction of urban–rural disparities in access to digital healthcare (7, 18).

## 5 Conclusion

An analysis of the current state of Peruvian Public Health through a review of the literature has provided insight into the current opportunities and constraints for employing Data Analytics as part of the Peruvian Public Health System. It was found that utilising Digital Health Technologies (DHT), Predictive Analytics (PA) and Interoperable Information Systems (IIS) could lead to increased efficiency of health care access and provision; increase the ability for effective and timely epidemiological surveillance of disease outbreaks; and ultimately help drive better quality and more predictive decisions regarding improving the overall healthcare outcomes of Peruvians and increase healthcare policy making decisions based on sound evidence. However, the evidence also suggests that the initiation of the technology components (DHT, PA and IIS) are not sufficient for long-term sustainability and performance improvement of the Peruvian Public Health System.

Institutional fragmentation, limited interoperability, unequal levels of digital infrastructure, governance limitations and continuing territorial inequalities affecting access to Health Care have been identified as some of the main barriers. In Peru, the co-existence of different healthcare subsystems makes it more difficult to integrate all digital health initiatives at the national level.

The paper adds to existing literature by offering a unique approach to analyzing how interoperability, governance, predictive analytics and equitable factors fit together as part of the public health digital transformation process in Peru. Future studies on understanding the institutional and technological factors affecting healthcare modernization within developing nations can benefit from this framework.

It is also important to acknowledge some of the limitations of this study. Most of the information used in this study comes from secondary sources such as research papers and reports from different institutions. Furthermore, there is a lack of empirical research related to publicly run Analytically Driven Public Health Systems specific to Peru. Therefore, in future work it would be beneficial to include additional types of empirical studies such as comparative original empirical studies, individual case studies, and quantitative assessment of digital health implementations within selected geographical regions of Peru.

The results indicate that there is no single answer or approach to the successes of public health analytics over time in Peru, but that the ability to build long-term sustainability requires growth through the combination of new technologies, continued cooperative efforts at the institutional level, continued improvements to the regulations governing public health analytics, continued workforce training in this area, as well as equitable policies for digital inclusion that will create pathways for healthcare systems to address their disparities and improve access to care for their citizens.
